# Plant-derived extracts and metabolic modulation in leukemia: a promising approach to overcome treatment resistance

**DOI:** 10.3389/fmolb.2023.1229760

**Published:** 2023-07-13

**Authors:** Cindy Mayerli Arévalo, Nataly Cruz-Rodriguez, Sandra Quijano, Susana Fiorentino

**Affiliations:** ^1^ Grupo de Inmunobiología y Biología Celular, Facultad de Ciencias, Pontificia Universidad Javeriana, Bogotá, Colombia; ^2^ Versiti Blood Research Institute, Milwaukee, WI, United States

**Keywords:** resistance, acute leukemias, metabolism, natural products, botanical drugs

## Abstract

Leukemic cells acquire complex and often multifactorial mechanisms of resistance to treatment, including various metabolic alterations. Although the use of metabolic modulators has been proposed for several decades, their use in clinical practice has not been established. Natural products, the so-called botanical drugs, are capable of regulating tumor metabolism, particularly in hematopoietic tumors, which could partly explain the biological activity attributed to them for a long time. This review addresses the most recent findings relating to metabolic reprogramming—Mainly in the glycolytic pathway and mitochondrial activity—Of leukemic cells and its role in the generation of resistance to conventional treatments, the modulation of the tumor microenvironment, and the evasion of immune response. In turn, it describes how the modulation of metabolism by plant-derived extracts can counteract resistance to chemotherapy in this tumor model and contribute to the activation of the antitumor immune system.

## 1 Introduction

Acute leukemias (ALs) are a group of malignant diseases of the hematopoietic system characterized by disordered proliferation and clonal expansion of immature precursors, resulting in bone marrow failure with blockage in cell differentiation processes (Swerdlow et al., 2017). ALs are classified, considering their morphological, immunophenotypic, cytogenetic, and molecular characteristics, into two large groups: acute myeloid leukemia (AML) and acute lymphoblastic leukemia (ALL). AML is characterized by the accumulation and blocked differentiation of progenitors of myeloid, monocytic, erythroid, or megakaryocytic lineage, and ALL instead arises from the transformation of B-cell (B-ALL) or T-cell progenitors (T-ALL) (Arber et al., 2016).

Although the use of various chemotherapy schemes for the treatment of ALs has achieved a high percentage of survival in developed countries, in Colombia its implementation has shown discouraging results, with complete remission rates of 45% in AML and 61% in ALL in the adult population (Combariza et al., 2007; Ballesteros-Ramírez et al., 2020a; Sossa et al., 2021). Treatment options for patients who do not achieve a complete remission are limited, as even with salvage therapy followed by allogeneic stem cell transplantation (HSCT), outcomes remain poor (Thol, 2018). For four decades, the mortality rates of patients with ALs have been the highest when compared to the rates of other groups of leukemias (Shallis et al., 2019).

The lack of response to current chemotherapeutics has been attributed to different resistance mechanisms, among them: 1) the acquisition of new mutations or genetic alterations (clonal evolution) (Hackl et al., 2017); 2) the presence of leukemia-initiating cells (LICs) remaining after chemotherapy (Yeung and Radich, 2017); 3) the autophagy as a cooperative mechanism for the stability of oncoproteins or as a cytoprotective against the cytotoxic effects induced by drugs (Auberger and Puissant, 2017); 4) the overexpression of ATP-binding cassette transporters (ABC transporters) that allow drug efflux (Marin et al., 2016); 5) the microbiota affecting the metabolism, toxicity, and efficacy of certain drugs (Wilkinson et al., 2018); 6) inherent patient factors such as body mass index and age (Li et al., 2017), 7) the microenvironment through soluble factors or direct interaction between leukemic blasts and mesenchymal or stromal cells (Shafat et al., 2017); and 8) which has recently been recognized as one of the hallmarks of cancer, can also contribute to the evasion of the antitumor immune response (Desbats et al., 2020).

Reprogrammed metabolic activities of tumor cells that support survival, maintenance, and drug response are related to altered bioenergetic pathways, enhanced macromolecule biosynthesis, and maintenance of the redox balance (DeBerardinis and Chandel, 2016; Vander Heiden and Deberardinis, 2017). The addition of therapies that modulate tumor metabolism to conventional chemotherapy has been emerging, (Luppi et al., 2018; Winer and Stone, 2019), and more therapeutic strategies are needed that achieve tumor metabolic modulation that can lead to the activation of the immune system and an accentuated anti-neoplastic effect. Then, our group focused on seeking anti-leukemic therapies based on plant-derived extracts that serve as the basis for the preparation of botanical drugs or phytomedicines (Urueña et al., 2008; Castañeda et al., 2012; Sandoval et al., 2016; Ballesteros-Ramírez et al., 2020b). The use of plant-derived extracts, due to their multiple active components, offers a multi-target mechanism of action, unlike many current therapies that follow the classic “one drug, one target” pharmacological dogma and have fewer toxic side effects, so they are more tolerable (Herranz-López et al., 2018). In addition, its main components can be combined with conventional chemotherapy to reduce the development of chemoresistance through the modulation of metabolism (Kumar and Jaitak, 2019).

In this review, we describe the intrinsic and extrinsic mechanisms that have been associated with alterations in glycolytic metabolism and mitochondrial function in chemoresistant leukemic cells and how natural products could reverse resistance with a metabolic and immunomodulatory approach.

## 2 Reprogramming glycolytic metabolism induces chemoresistance in ALs

The final product of glucose oxidation can be lactate or carbon dioxide (CO_2_) (via mitochondrial respiration). Tumor cells, regardless of oxygen availability, increase glucose consumption and produce large amounts of lactate, which is known as the Warburg effect (Icard et al., 2018; Liu et al., 2021; Marcucci and Cristiano, 2021). The increase in the glycolytic pathway is advantageous for tumor cells because: first, it promotes uncontrolled proliferation due to biomass generation; second, it is a fast way to produce adenosine triphosphate (ATP), even more than oxidative phosphorylation (OxPhos); and third, it prevents damage from oxidative stress, directly by reducing mitochondrial respiration and therefore the generation of reactive oxygen species (ROS), and indirectly by the production of nicotinamide adenine reduced dinucleotide phosphate (NADPH) through the pentose pathway (PPP), which maintains glutathione (GSH) in a reduced state, the main non-protein thiol that acts as an intracellular redox regulator (Marcucci and Cristiano, 2021). Some intrinsic and extrinsic factors related to increased glycolysis described below are associated with chemoresistance mechanisms in various tumor models, including ALs (Supplementary Table S1).

### 2.1 ALL and AML cells resistant to chemotherapy and increase glycolysis

Most of the studies in cell lines and primary samples derived from myeloid and lymphoid leukemias demonstrate a relationship between the increase in the glycolytic pathway and resistance to different types of drugs, such as anthracyclines (daunorubicin, -DNR-, doxorubicin, -DOX-, and idarubicin, -IDA-), some glucocorticoids such as prednisolone, and tyrosine kinase inhibitors (TKIs) such as imatinib, among others.

In DNR-resistant cell lines such as HL60/DNR and CEM/R2, of myeloid and T-lymphoid origin, respectively, a higher glucose demand has been described together with a lower glutamine dependence and a lower rate of fatty acid oxidation compared with their non-resistant counterparts (Stäubert et al., 2015). Similarly, myeloid cell lines (K562-r and LAMA84-r) with aberrant expression of Breakpoint Cluster Region-Breakpoints in the Abelson (ABL)1 fusion (BCR-ABL1) and resistance to imatinib maintain a highly glycolytic metabolic phenotype with elevated lactate production (Kominsky et al., 2009). On the other hand, the TEX cell line, which mimics the characteristics of AML and LICs, is resistant to tigecycline (an antibiotic that induces an antitumor effect by inhibiting the translation of mitochondrial proteins) (Xu et al., 2016), and it has an increased glycolytic rate (Jhas et al., 2013). Importantly, in some B-ALL and T-ALL cell lines and cells derived from patients with B-ALL, reduction of the glycolytic rate using 2-deoxy-D-glucose (2-DG), lonidamine (LND), or 3-bromopyruvate (3-BrPA), can restore the sensitivity of these cells to glucocorticoids (Hulleman et al., 2009; Gu et al., 2017); and the combined treatment of a glycolytic inhibitor in conjunction with the silencing of anti-apoptotic proteins such as myeloid cell leukemia sequence 1 protein (MCL-1) can enhance the antitumor activity in cells resistant to prednisolone (Ariës et al., 2013).

In contrast to the above, a single publication by Herst et al. (2011) using primary AML cells describes that patients with highly glycolytic blasts had higher overall survival rates and longer rates of first complete remission compared to patients who had blasts with moderate glycolytic rate. Likewise, leukemic blasts that had a high glycolytic level were more susceptible to the anti-tumor activity of all-trans retinoic acid (ATRA) and arsenic trioxide (ATO). These data show us that tumor cells require homeostatic mechanisms to avoid scenarios of accumulation or depletion of secondary metabolites that can be toxic and that an excessive glycolytic flux can represent an unfavorable event for their survival and adaptation to different stress conditions. However, the direct relationship between the increase in glycolysis and chemoresistance in leukemic cells must be studied in depth at the mechanistic level.

### 2.2 Regulation of increased glycolytic flux: intrinsic mechanistic factors

**FIGURE 1:**
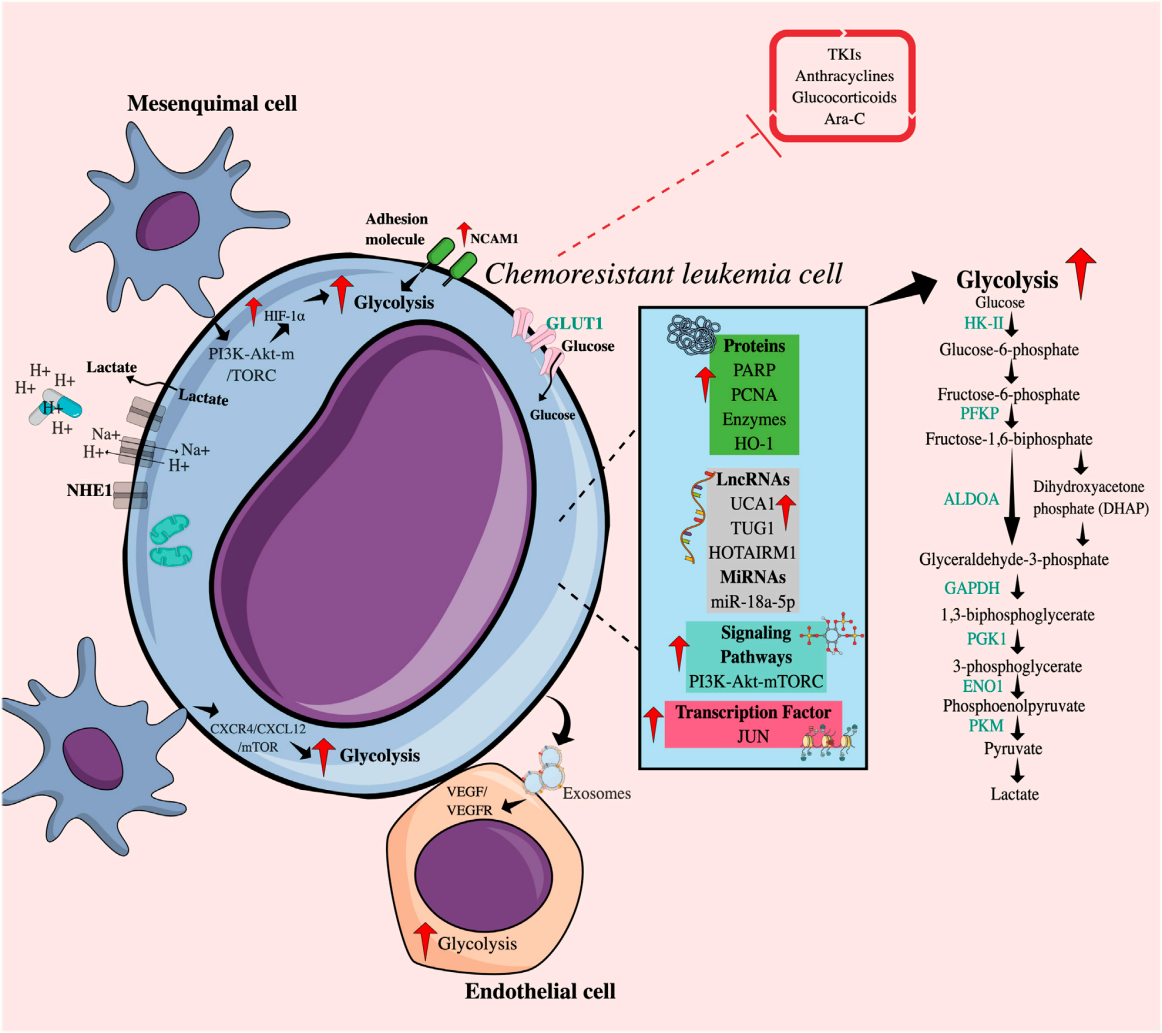
Intrinsic and extrinsic mechanisms related to increased glycolytic flux in chemoresistant leukemia cells. Intrinsically, chemoresistant leukemia cells overexpress cytoplasmic proteins, enzymes, RNA molecules, transcription factors, and receptors and present alterations in signaling pathways that favor an increase in glycolytic flow. Additionally, the direct or indirect interaction between chemoresistant leukemia cells and various cell populations of the tumor microenvironment, such as mesenchymal and endothelial cells, promotes glucose uptake achieved by tumor cells through the PI3K-mTORC pathway or the CXCR4/CXCL12 axis. The release of exosomes containing VEGF-RVEFG can also increase glycolysis in endothelial cells. Taken together, all these factors may regulate resistance to drugs including TKIs, anthracyclines, Ara-C, and glucocorticoids. TKIs, Tyrosine kinase inhibitors; Ara-C, Cytarabine; HIF-1, Hypoxia-induced factor; GLUT1, Glucose transporter 1; NCAM1, Neural cell adhesion molecule 1; NHE1, Na (+)/H (+) antiporter; HK-II, Hexokinase II; PFKP, Phosphofructokinase; GPI, Glucose phosphate isomerase; ALDOA, Aldolase A; GAPDH, Glyceraldehyde-3-phosphate dehydrogenase; PGK1, Phosphoglycerate kinase; ENO1, Enolase 1; LDHA, Lactate dehydrogenase A; PKM, Pyruvate kinase 2; HO-1, Heme oxygenase-1; PARP, Poly (ADP-ribose) polymerases; PCNA, Proliferating cell nuclear antigen; LncRNAs, Long non-coding RNAs; microRNAs, Short non-coding RNAs; UCA1, Associated with urothelial carcinoma; TUG1, Taurine upregulated gene 1; HOTAIRM1, Myeloid-specific antisense intergenic RNA HOX transcript 1.

Different intrinsic factors have been established that influence an improvement in the glycolytic pathway in chemoresistant leukemia cells (Figure 1). Genetic alterations that involve mutations in oncogenes and/or tumor suppressor genes lead to deregulated signaling pathways and consequently to the aberrant expression of transcription factors, enzymes, transporters, proteins (nuclear and cytoplasmic), and regulatory molecules of glucose metabolism (Lo Presti et al., 2020; Di Martino et al., 2021).

The phosphoinositide 3-kinase-Akt-mammalian target of rapamycin (PI3K-Akt-mTOR) signaling pathway, commonly activated in cancer, including AML, contributes to the maintenance of glycolysis by translational and post-translational regulation of metabolic enzymes (Hoxhaj and Manning, 2020; Nepstad et al., 2020). Ryu et al. (2019) established that AKT activation by phosphatidylinositol 3,4,5-trisphosphate 3-phosphatase and dual-specificity protein phosphatase (PTEN) deficiency, a direct PI3K antagonist, maintained a refractory state of cells to treatment with cytarabine (Ara-C) and IDA through the improvement of glycolysis in the myeloid lines HL60 and KG1α.

A master transcription factor in the regulation of glycolysis is hypoxia-inducible factor 1 α (HIF-1α) because it promotes the transcription of glucose transporters, such as glucose transporter 1 (GLUT1), and glycolytic enzymes, such as hexokinase II (HK-II), and pyruvate kinase M2 (PKM2) (Yu et al., 2017). An increase in the expression levels of GLUT1, HIF1-α, and HK-II mRNA has been reported in primary AML cells from non-responders and HL60/DNR cells (Jhas et al., 2013; Song et al., 2014; Song et al., 2016). Also, recently, Valin et al. (2022) reported JUN, an oncogenic transcription factor, as a regulator of glycolytic metabolism in AML, since it increases the expression of hexokinase I (HKI) and HKII, glucose phosphate isomerase (GPI), phosphofructo-1-kinase (PFKP), aldolase A (ALDOA), glyceraldehyde-3-phosphate dehydrogenase (GAPDH), phosphoglycerate kinase (PGK1), enolase 1 (ENO1), and PKM. Similarly, in breast cancer, the c-JUN family of proteins has been associated with resistance to cisplatin (Xia et al., 2013).

Another molecule that promotes glycolysis is the proliferating cell nuclear antigen (PCNA), which is a nuclear protein synthesized in the early G1 phase and in the S phase of the cell cycle and is considered a marker of the proliferation index in some neoplasms (Cardano et al., 2020). It has been shown that the interaction between PCNA and nicotinamide phosphoribosyl transferase (NAMPT), an enzyme that participates in the rescue pathway for the generation of the nicotinamide adenine dinucleotide (NAD+) cofactor, can coordinate the increase in glycolysis, favoring the survival of DNR-resistant HL60 cells (Ohayon et al., 2016).

On the other hand, the enzyme heme oxygenase-1 (HO-1) catalyzes the degradation of the heme group, causing carbon monoxide (CO), biliverdin, and iron, where the heme group is a critical component of multiple hemoproteins involved in glucose metabolism and of lipids and proteins. Interestingly, elevated levels of HO-1 have been described in primary myeloid cells and HL60 cells resistant to Ara-C and DNR. HO-1 inhibition abrogates the expression of HIF-1α, and GLUT1, improving the sensitivity to the two drugs, apparently by decreasing the glycolysis (Zhe et al., 2015). Furthermore, HO-1 is involved in the generation of a resistant profile in patients with myelodysplastic syndrome (MDS) who can progress to AML (He et al., 2019). Also, the increased expression of the adhesion protein NCAM1 (Neural Cell Adhesion Molecule 1) in K562 BCR-ABL^+^ myeloid cells increases the IC_50_ for dasatinib (Sasca et al., 2019). Knockdown of NCAM1 decreases the expression of genes involved in glucose metabolism, and the reduction in their expression sensitizes NOMO shNCAM_3 leukemic cells to treatment with Ara-C in a murine NSG (NOD *scid* gamma mouse) model.

Poly (ADP-ribose) polymerases (PARP) family of polymerases is made up of proteins involved in many cellular processes, including DNA repair and apoptosis. Particularly, PARP14 can promote glycolysis in different tumor models, such as human hepatocellular carcinoma (HCC), by maintaining reduced PKM2 activity (Iansante et al., 2015); on the other hand, a close relationship between c-myc and AKT has been reported in B lymphomas (Cho et al., 2011) and AML through the NF-κB/HIF-1α axis (Zhu et al., 2022). Interestingly, the use of Niraparib, a PARP1/2 inhibitor, decreases resistance to ATO and hypomethylating agents (azacytidine and decitabine) in tumor promyelocytes (Giansanti et al., 2021).

Deregulation of LncRNAs and microRNAs (long and short non-coding RNAs, respectively), considered epigenetic regulators, contributes to therapeutic resistance through the regulation of energy metabolism (Agbu and Carthew, 2021; Taghvimi et al., 2022). The LncRNA associated with urothelial carcinoma (UCA1) is overexpressed in AML cells from patients who did not respond to DOX treatment and in the HL60/DNR cell line (Liu et al., 2014). UCA1 promotes glycolysis by inhibiting the action of mRNA125a, which usually functions as a tumor suppressor but also appears to be involved in blocking HK-II (Sun et al., 2017), which in turn could participate in the stabilization of HIF1-α (Zhang et al., 2018). Also, thanks to its oncogenic role, the increase in the expression of the LncRNA TUG1 (taurine-upregulated gene 1) is accompanied by the increase in the expression of the mRNAs of HK-II and PKM2 in HL60 cells resistant to doxorubicin (HL60/DOX) (Chen L. et al., 2019a). Another LncRNA, HOTAIRM1 (HOX transcript antisense intergenic RNA myeloid-specific 1), involved in myeloid lineage maturation and overexpressed in AML, has been compromised by resistance to Ara-C. Its deletion has been reported to improve drug activity and decrease glucose consumption and therefore lactate production, mainly mediated by the reduction of PFKP in HL60/WT and THP-1/WT cells (Chen et al., 2020).

Particularly in K562 cells, the expression level of miR-18a-5p in DOX-resistant cells is lower compared to K562 cells and normal lymphocytes, and strikingly, miR-18a-5p can inhibit the expression of HIF-1α and, in turn, induce a reduction in the production of pyruvate, ATP and affect the expression of GLUT1, HK-II, PKM2, and lactate dehydrogenase A (LDHA) (Wu et al., 2021).

### 2.3 Regulation of increased glycolytic flux: extrinsic mechanistic factors

In the bone marrow microenvironment, there are cellular and non-cellular components that influence the metabolic reprogramming of leukemia cells and can be considered external factors (Jiang and Nakada, 2016). Contact between mesenchymal cells (MSC) and/or endothelial cells (EC) and leukemic cells improves tumor survival in the presence of different chemotherapeutic agents (Boutin et al., 2020; Okamoto et al., 2022). Culturing primary B-ALL cells or Reh lymphoid cell line with bone marrow-derived MSCs under hypoxic conditions induces a higher expression of HIF-1α and therefore an acquisition of the glycolytic phenotype of leukemic cells, which in part is due to signaling-regulated by the AKT/mTOR pathway induced by stromal cells. Inhibition of mTOR using everolimus, an antiproliferative agent, reduces the expression of HIF-1α, decreases the glycolytic rate, and partially restores the sensitivity of ALL cells to vincristine under co-culture and hypoxic conditions (Frolova et al., 2012). Also, the interaction with stromal cells increases the glycolytic flux of AML cells from patients through the CXCR4/CXCL12/mTOR axis (Braun et al., 2016), and the use of selective inhibitors of this axis represents an opportunity to block MSC-mediated chemoresistance (Braun et al., 2016). In AML, leukemic cells have been reported to secrete VEGF/VEGFR-containing exosomes that induce glycolysis in endothelial cells -HUVECs-, leading to vascular remodeling and the acquisition of chemoresistance (Wang et al., 2019).

On the other hand, the Warburg effect implies an increase in the production of lactic acid and, therefore, an acidic microenvironment. Tumor cells express several families of pH regulators in the plasma membrane to protect themselves, such as NHE1 (Sodium-hydrogen antiporter 1), which exports H^+^ and contributes to the decrease in extracellular pH. Acidification in the microenvironment plays an immunosuppressive role and mediates the protonation of drugs, affecting their cell permeability (Altaf et al., 2017; de la Cruz-López et al., 2019). In T-ALL, increased NHE1 activity may promote resistance to DOX and imatinib (Man et al., 2014).

### 2.4 Association between increased glycolysis with clinical response and survival rates in ALs

Despite the strong evidence that glycolytic metabolism can promote chemoresistance in primary leukemic cells from non-responders and/or multiresistant cell lines, recent advances in metabolomics platforms have allowed further investigation into tumor metabolism at the systemic level in patients with acute leukemia (Grønningsæter et al., 2019; Kim et al., 2021), however, only a few studies have focused on the impact of glycolytic alterations on response to chemotherapy treatment, with mixed results. An increase in circulating serum glucose (α-glucose and β-glucose) in patients with different subtypes of AML—Including leukemias with monocytic differentiation characterized by having a poor prognosis—In comparison with healthy controls has been reported (Duan et al., 2022). In contrast, in plasma samples of bone marrow from pediatric ALL patients with positive minimal residual disease (MRD), a decrease in glucose and a higher concentration of other metabolites related to glycolysis, the PPP pathway, and the tricarboxylic acid (TCA) cycle were observed (Schraw et al., 2019). These results demonstrate the functional compartmentalization of glucose and the ability of tumor cells to influence systemic glucose regulation (Ye et al., 2018). Likewise, Chen et al. (2014) managed to identify a serum metabolic signature related mainly to the glycolysis pathway, where the decrease in glycerol-3-phosphate (glycerol-3P) and lactate, together with the increase in 2-hydroxyglutarate (2-HG), 2-oxoglutarate, and pyruvate, would be negatively related to the overall survival of patients with AML with a normal karyotype; while decreased citrate levels would positively impact, evidencing the clinical utility of the identification of energetic metabolic biomarkers. However, taking into account that the global metabolome of a patient is a complex, specialized metabolic network at different levels (Schmidt et al., 2021) and that it is not only influenced by the glycolytic metabolism of tumor cells, but the focus of metabolism studies in cancer with clinical utility must also have a comprehensive vision.

## 3 Chemoresistance-induced mitochondrial function in ALs

Mitochondria allow metabolic adaptation to various stress conditions as they are an integrating center of important cellular processes that include energy production through OxPhos, redox signaling, anabolic and catabolic reactions, epigenetic regulation, cell self-renewal and differentiation, initiation, and run-time programmed cell death (PCD) (Basak and Banerjee, 2015; Guerra et al., 2017; Bokil and Sancho, 2019; Grasso et al., 2020); and particularly in leukemias, they have emerged as a determinant for the progression and development of cancer as well as the response to chemotherapeutics (Basak and Banerjee, 2015). Interestingly, several studies have shown that AML and B-ALL cells have increased respiratory activity, increased mitochondrial mass, and significantly higher mitochondrial DNA (mtDNA) content than normal CD34^+^ cells and peripheral blood mononuclear cells (PBMCs) (Panina et al., 2019; Jain et al., 2022). However, they show a lower energy reserve capacity and reduced activity of complexes III, IV, and V, which could explain why they are more sensitive to mitochondrial damage than normal cells or other tumor models, such as ovarian cancer (Panina et al., 2019). In addition, synergistic and selective cytotoxicity has been reported in AML cell lines by combining components that target mitochondria (mitocans) with glycolytic inhibitors, TKIs, or microtubule destabilizers (Panina et al., 2020). Added to the above, cells with low levels of ROS, within which LICs could be, are unable to use glucose when mitochondrial respiration is inhibited (Lagadinou et al., 2013). For all the above, mitochondrial function is essential for the survival of leukemic cells and is currently a highly explored therapeutic target.

### 3.1 Mitochondria as a promoter of chemoresistant AML and ALL cells

**FIGURE 2:**
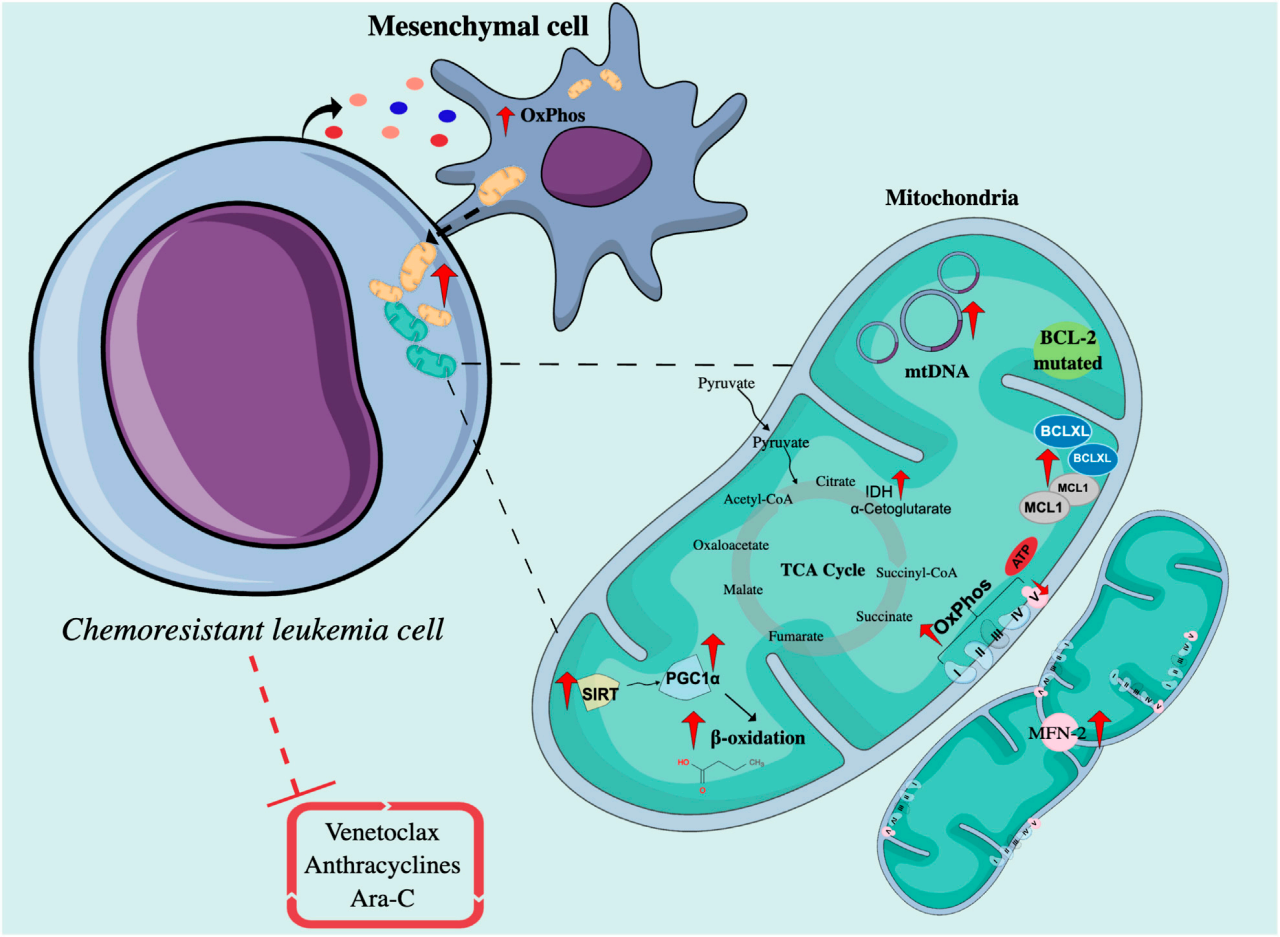
Intrinsic and extrinsic mechanisms related to mitochondrial function in chemoresistant leukemia cells. Chemoresistant leukemia cells exposed to drugs such as Venetoclax, Ara-C, and anthracyclines interact with mesenchymal cells through mitochondrial transfer to increase OxPhos and ATP production. On the other hand, the mitochondria of resistant cells are characterized by the upregulation of proteins involved in the regulation of apoptosis, mitochondrial biogenesis, and fatty acid metabolism, and present a higher number of mtDNA copies. OxPhos, Oxidative phosphorylation; Ara-C, Cytarabine; TCA cycle, Cycle of tricarboxylic acids; MFN-2, Mitofusin 2; SIRT, Sirtuins; PGC1α, PPARγ coactivator-1α; IDH, Isocitrate dehydrogenase; mtDNA, Mitochondrial DNA; BCL-2, B-Cell Leukemia/Lymphoma 2; Bax, Bcl-2-associated X protein; Bak, Bcl-2 homologous antagonist/killer; MCL1, Myeloid cell leukemia sequence 1 protein.

Various publications have described the role of mitochondrial activity in resistance or sensitivity to chemotherapy treatment in ALs. Most of the reports currently described show that resistant leukemic cells have enhanced mitochondrial activity, while other works suggest that impaired or stable mitochondrial activity could be related to resistance. The association between mitochondrial activity and low sensitivity to anthracyclines, Ara-C, and Venetoclax has been documented in cell lines and primary leukemia cells and confirmed using *in vivo* models (Farge et al., 2017) (Figure 2).

Regarding improved mitochondrial activity, AML cells persistent to treatment with Ara-C in an NSG mouse model presented a higher rate and a genetic signature associated with OxPhos, accompanied by an increase in mitochondrial mass, membrane potential, and ROS production (Farge et al., 2017). Through the use of metabolic sensors, in B-ALL, it was shown that cells resistant to Ara-C had greater mitochondrial respiration capacity (Chen et al., 2021). Several reports have shown that by inhibiting mitochondrial processes such as the oxidation of fatty acids, the activity of the electron transport chain (ETC), the replication of mtDNA, or the synthesis of mitochondrial proteins, the sensitivity to Ara-C increases *in vitro* and *in vivo* (Farge et al., 2017), and the proliferation of primary AML cells have slightly decreased (Vitkevičienė et al., 2019).

On the other hand, regarding stable or impaired mitochondrial activity, in Jurkat rho zero (ρ0) T lymphoid cells (cells without mitochondria), there is greater resistance to treatment with bleomycin, an antineoplastic antibiotic, compared to Jurkat cells with mitochondria (Yeung et al., 2015), reflecting a susceptibility to mitochondrial damage in these cells. And also, interestingly, Henkenius et al. (2017) reported that resistant HL60 and MV4-11 cells maintain stable mitochondrial activity after exposure to different concentrations of Ara-C and sorafenib, compared to sensitive HL60 cells, in which their mitochondrial activity is affected.

### 3.2 Intrinsic and extrinsic mechanistic factors of mitochondrial function mediating chemoresistance in ALs cells

There are also intrinsic and extrinsic factors that affect mitochondrial function and, therefore, mediate therapeutic sensitivity. Among the intrinsic factors are the sirtuins (SIRTs), a family of NAD^+^-dependent lysine deacetylases, some of which are exclusively expressed in mitochondria (SIRTs 3, 4, and 5) (Jaiswal et al., 2022). These enzymes mediate the response to cellular stress, inducing metabolic changes by regulating the activity of proteins such as PGC1α (PPARγ coactivator-1α) (Chalkiadaki and Guarente, 2015), which is a master in the regulation of mitochondrial metabolism and an activator of genes associated with the oxidation of fatty acids (Mattes et al., 2019). In AML, SIRT3 activity has been implicated in chemoresistance to Ara-C by regulating OxPhos in the myeloid cell line MV4-11 (Ma et al., 2019). In addition, it has been described that many AML patient samples are highly dependent on SIRT5. Genetic or pharmacological SIRT5 inhibition impairs *in vitro* transformation of mouse hematopoietic cells by several myeloid oncogenes, including MLL-AF9, and attenuates *in vivo* leukemogenesis. SIRT5 knockdown or pharmacological inhibition with NRD167 is associated with reduced OXPHOS, reduced GSH levels, and increased mitochondrial superoxide, suggesting that AML cells depend on SIRT5 to maintain redox homeostasis (Yan et al., 2021).

Another important enzyme in mitochondrial function is ATP synthase, a transmembrane enzyme that catalyzes the synthesis of ATP from ADP, a phosphate group, and the energy supplied by the flow of H^+^ supplied by the coenzymes NADH and reduced flavin adenine dinucleotide (FADH_2)_. It is made up of two subunits: the Fo subunit, a component that crosses the inner mitochondrial membrane, and the F1 subunit, which protrudes into the mitochondrial matrix. The decrease in the expression of the ATP-F1-β subunit, probably due to epigenetic changes related to hypermethylation of the gene, has been reported in cells from refractory or relapsed AML patients and has been confirmed in DOX-resistant HL60 myeloid lines and DNR (Xiao et al., 2013; Song et al., 2016; Yang et al., 2016).

Mitochondrial fusion and fission are two opposite processes that go according to cellular metabolic requirements. Fusion allows mitochondria to connect with each other to form networks or fragments and is commonly associated with increased ATP production and protection against autophagy; on the contrary, fission allows mitochondria to constantly divide and is mainly related to apoptosis, facilitating the segregation of mtDNA in mitosis and eliminating defective mitochondria (Genovese et al., 2021). The survival of T-ALL cells after DOX treatment was correlated with the expression of Mitofusin-2 (MFN-2)—A GTPase protein that mediates mitochondrial fusion—With an increase in the expression of CTE complexes as well as a proportional increase in oxygen consumption rate (OCR), and increased sensitivity to DOX was evident upon inactivating MFN-2 by CRISPR (Decker et al., 2020).

The anti-apoptotic protein Bcl-2 homologous antagonist/killer (BCL-2), overexpressed in some subtypes of B-cell lymphomas, regulates mitochondrial function and mediates therapeutic resistance by inhibiting the oligomerization of the pro-apoptotic Bcl-2 homologous antagonist/killer (Bak) and Bcl-2-associated X protein (Bax) proteins and by facilitating the import of GSH and complex IV subunits to mitochondria (Mattes et al., 2019). The dependence of BCL-2 activity has been demonstrated in both chemosensitive and chemoresistant myeloblasts but not in normal hematopoietic stem cells (HSCs) (Vo et al., 2012); therefore, the use of BCL-2 inhibitors has recently emerged, especially the use of venetoclax in the treatment of AML (Griffioen et al., 2022). Despite its potential *in vivo*, factors such as the upregulation of fatty acid oxidation (FAO) (Stevens et al., 2020), mutations in BCL-2, overexpression of the anti-apoptotic proteins MCL1 or B-cell lymphoma extra-large (Bcl-xL) (Saliba et al., 2021), or the OPA1 (Mitochondrial Dynamin Like GTPase) fusion protein have been described as mediators of resistance to venetoclax (Chen X. et al., 2019b).

The enzymes isocitrate dehydrogenases (IDH) produce NADPH by metabolizing isocitrate to α-ketoglutarate (α-KG), which is an important factor in the TCA cycle. In AML, IDH mutations lead to the consumption of NADPH by converting isocitrate to 2-HG, considered an oncometabolite. The accumulation of 2-HG triggers the inhibition of α-KG-dependent enzymes, such as TET2 (Tet methylcytosine dioxygenase 2) demethylase, and consequently alters gene expression by favoring DNA hypermethylation (Raimondi et al., 2022). The improvement in mitochondrial oxidative metabolism has been associated with resistance to the use of IDH inhibitors and the concomitant use of OxPhos inhibitors improves the efficacy of treatment against IDH mutated *in vivo* in AML (Stuani et al., 2021).

Regarding external factors, microenvironment-induced tumor reprogramming also affects mitochondrial function. In AL, the transfer of mitochondria between leukemic cells and MSCs as a mechanism of tumor protection has been reported (Polak et al., 2015; Moschoi et al., 2016; Wang et al., 2018). Transfer inhibition using metformin improves the chemosensitivity of AML cells cocultured with MSCs to Ara-C (You et al., 2022). In fact, Ara-C increases mitochondrial transfer from MSCs to AML cells triggered by OxPhos inhibition, favoring tumor progression (Saito et al., 2021). Zhang et al. (2022) described that OCI-AML3 myeloid cells induce adipogenic differentiation in MSCs and reduce osteoblastic differentiation (Figure 2). This alteration is accompanied by a metabolic change from glycolysis to a more oxidative form given by a decrease in phosphoglycerate mutase 1 (PGAM1), ALDOA, LDHA/B, and an increase in succinate dehydrogenase (SDH) A/B/C/D from the ADP-forming beta subunit of succinate-CoA ligase (SUCLA2), aconitase 2 (ACO2), PDK, and pyruvate dehydrogenase phosphatase (PDP), among others.

### 3.3 Association between mitochondrial function and clinical response and survival rates in ALs

In AML, patients with lower rates of remission and overall survival have a proteogenomic profile characterized by high expression of mitochondrial proteins and a more complex I-dependent respiration (Jayavelu et al., 2022). Additionally, Nan et al. (2022) in a group with high-risk AML identified an increased mitochondrial gene signature: electron transfer flavoprotein subunit beta (ETFB), carnitine palmitoyl transferase 1A (CPT1A), 4-Hydroxyphenylpyruvate Dioxygenase Like (HPDL), and isocitrate Dehydrogenase NAD^+^ 3 Catalytic Subunit Alpha (IDH3A) (Jiang et al., 2022). Likewise, patients with mutations in CTE complexes have worse overall survival compared to those without mutations (Silkjaer et al., 2013). Also, a proteomic study on primary AML cells derived from patients in their first relapse shows enrichment of mitochondrial ribosomal proteins (Mitochondrial Ribosomal Protein L21 -MRPL21-, Mitochondrial Ribosomal Protein S33 -MRPS3-), and CTE proteins: Translocase of Inner Mitochondrial Membrane Domain Containing 1 (TIMMDC1), a chaperone protein involved in the assembly of Complex I, and Mitochondrial Import Inner Membrane Translocase subunit (Tim8B TIMM8B), a chaperone that participates in the import and insertion of some transmembrane proteins in the inner mitochondrial membrane (Aasebø et al., 2020). In addition, patients treated with chemotherapy show lasting changes in the expression of genes involved with OxPhos activation, which can be explained because tumor cells, after chemotherapy-induced damage, increase their demand for ATP for repair processes (Vellinga et al., 2015). Therefore, these studies in patients support the idea that enhanced mitochondrial metabolism is associated with a poor prognosis; however, further studies are needed to elucidate the role of mitochondria in chemoresistance in acute leukemia.

## 4 Relationship between altered energy metabolism and the immune system

Malignant transformation is accompanied by changes in cell metabolism that have an impact on immune system function, reducing the control of cancer cells (O’Sullivan et al., 2019; Patel et al., 2019). These alterations include competition for substrates, the abundant release of bioactive metabolites, and microenvironmental metabolic remodeling that favors the induction or survival of subsets of tolerogenic cells (Mougiakakos, 2019). Modulation of tumor metabolism can improve immune cell activation, allowing an efficacious antitumor response, which achieves an effective therapeutic approach against ALs.

Increased glycolysis has been associated with inflammatory effector phenotypes in a variety of activated immune cells. In contrast, mitochondrial oxidation programs are associated with memory, suppressor, or wound-healing immune cell phenotypes. Each subset of immune cells has been shown to have distinct metabolic requirements. Thus, although T cells, macrophages, and dendritic cells (DC) each have significant plasticity to engage in other metabolic pathways for energy generation, survival, and proliferation, these changes alter or impair immune function. These alterations in cell function are due to the needs of each immunological subset for specific metabolites, signaling intermediates, or epigenetic modifications mediated by metabolite levels. As metabolic intermediaries change, cell differentiation is not adequately induced, or stress response pathways feedback to restrict or alter the ultimate fate of cells (Andrejeva and Rathmell, 2017).

### 4.1 Tumor glycolytic metabolism and immune response

The metabolites and waste products excreted by tumor cells (lactate, CO_2_, H^+^, NH4^+^, nitric oxide, butyrate, polyamines, and ROS, among others) establish acidification of the microenvironment, that is, compatible with cell proliferation and dissemination, promoting several processes such as decreased cell adhesion, angiogenesis, and mesenchyme-epithelial transition (MET) in other tumor models.

Tumor cells can evade the surveillance of the immune system by secreting lactate. This extracellular metabolite generates acidification of the medium that reduces the pH inside immune cells, affecting various signaling pathways and inhibiting the activation and proliferation of CD4^+^, CD8^+^ T cells, NK cells, and DCs. In models of solid tumors, it has been described that the increase in lactate stimulates the polarization of resident macrophages to M2-type macrophages (immunosuppressants), promotes angiogenesis, and stimulates the production of hyaluronic acid by fibroblasts that can contribute to tumor invasion (Hirschhaeuser et al., 2011; Pavlova and Thompson, 2016; de la Cruz-López et al., 2019). It has been described that ROS and HIF can promote a “reverse Warburg effect” in cancer-associated fibroblasts (CAFs), which release lactate through the MCT4 (proton-linked monocarboxylate transporter) receptor, which is captured by tumor cells through MCT1, to be converted to pyruvate and metabolized at the mitochondrial level (Icard et al., 2018). In AML, it has been described that lactate could contribute to the induction of regulatory T lymphocytes and suppressor myeloid cells. (Mougiakakos, 2019). Cancer cells’ greed for glucose inactivates effector T cells and DCs, whereas programmed cell death ligand-1 (PD-L1) expressed by tumor cells stimulates glycolysis and the Akt pathway in cancer cells, thereby helping maintain its proliferation (Jayavelu et al., 2022).

### 4.2 Mitochondrial function and immune response

Mitochondria influence immunosurveillance through intrinsic and extrinsic mechanisms in cancer cells. On the one hand, mitochondria are the source of many danger signals released by dying cancer cells, and these signals are crucial for the DCs activation (Porporato et al., 2018) and tumor-associated macrophages (TAMs) (Icard et al., 2018), as well as the concentrations of succinate and citrate. M1 macrophages, which have a proinflammatory and anticancer role, are dependent on glycolysis and an abnormal TCA cycle that leads to citrate and succinate accumulation. Citrate accumulation increases the production of the main mediators of acute inflammation (nitric oxide, ROS, and prostaglandins), while succinate induces the production of IL-1β, a central molecule in inflammation (Icard et al., 2018).

Furthermore, mitochondrial metabolism is involved in many functions linked to cancer immunity, including (but not limited to) the activation of inflammasomes, the establishment of protective immunological memory, tumor subset differentiation, and macrophage-specific activity (Porporato et al., 2018). Mitochondria serve as sources of molecules that activate inflammatory pathways as well as signaling platforms to propagate these signals (Giampazolias and Tait, 2016).

2-HG has also been implicated in tumor immune evasion. 2-HG transport into T cells is facilitated by the sodium-dependent dicarboxylate transporter (SLC13A3), altering its effector function and cell proliferation. In addition, it interferes with the activation of activated T-cell nuclear factor 1 (NFATC1), a key transcription factor for activated T-cell function, which is linked to ATP deficiency. This oncometabolite also acts at the biochemical level in T cells by inhibiting ornithine decarboxylase, an enzyme that participates in the biosynthesis of polyamines (such as putrescine), molecules necessary for the proper functioning of T cells. This represents a self-perpetuating effect since putrescine can antagonize 2-HG, suppressing cell proliferation. 2-HG has also been shown to inhibit CD12 expression on DC and inhibit CXCL-10 secretion, thus preventing T-cell recruitment. In naive T cells, 2-HG is associated with the destabilization of HIF-1α, preservation of OxPhos, and increased differentiation of CD4^+^/CD25^+^/FOXP3^+^ regulatory T cells. This proceeds at the expense of differentiation into Th17 cells. Finally, 2-HG, through the stimulation of NF-kB, induces cell proliferation in a stromal niche for AML cells and, at relatively low concentrations, promotes fibroblast proliferation (Ježek, 2020).

## 5 Modulation of energy metabolism and activation of the immune system as targets of natural products

Substantial studies highlight the antineoplastic effect of natural products in addition to their use in the development of new drugs (Newman and Cragg, 2016; Sarwar et al., 2018), suggesting their great potential to become a coadjuvant alternative for the ALs treatment. According to their chemical composition, natural products can be subclassified into: alkaloids, carotenoids, nitrogen-containing compounds, organosulfur compounds, and phenolic compounds, the latter being the group that has the greatest *in vitro* effect on AML cells, perhaps because they are one of the substances more widespread among plants (Hwang et al., 2019).

Currently, there are natural products that have been used in the treatment of ALL and AML, such as vincristine, an alkaloid derived from *Catharanthus roseus*, and etoposide, a semisynthetic derivative of podophyllin obtained from *Podophyllum* (Lucas et al., 2010). In *in vitro* assays, there are various plants that show a cytotoxic effect on AML cells through different mechanisms (Hwang et al., 2019). Interestingly, the potentiation of the antineoplastic effect has been published when natural products and conventional chemotherapeutics are used in combination. In AML, there is a synergy between Ara-C and resveratrol. The latter can act by inhibiting the CTE complex III, affecting the pool of dNTPs, and reducing the proliferation of leukemic cells (Horvath et al., 2005).

**FIGURE 3:**
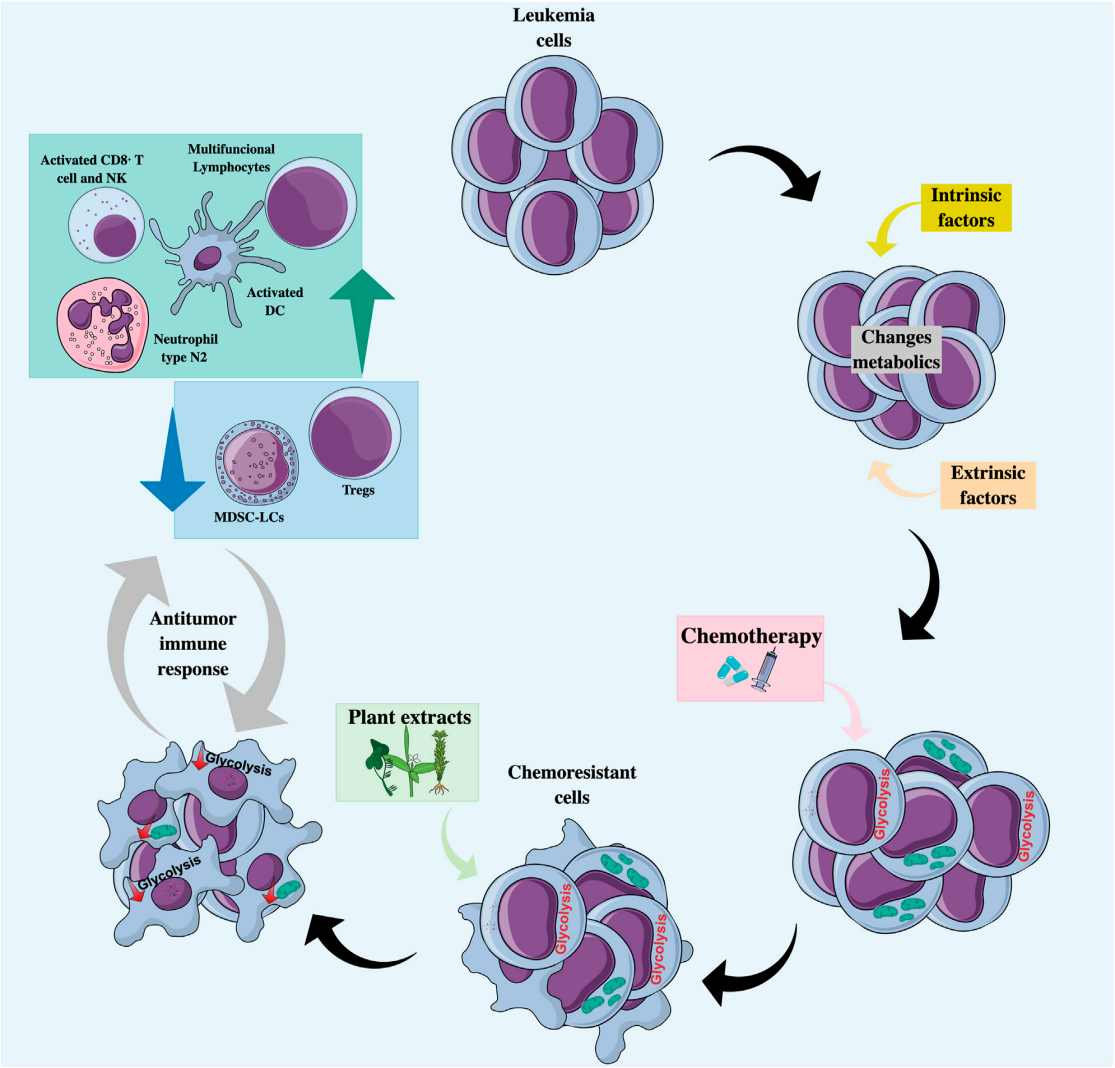
Natural products as co-adjuvant therapy in acute leukemia with a metabolic and immunomodulatory approach. Treatment with chemotherapeutic agents may favor the selection of chemoresistant clones characterized by an increase in glycolytic flux or an increase in mitochondrial activity. Combined treatment with natural products can lead to the regulation of tumor metabolism by decreasing glycolysis and/or altering mitochondrial function, making cells more sensitive to cell death, and promoting the recognition and elimination of tumor cells by cells of the immune system. DC, Dendritic cells; MDSC-LCs, Myeloid-derived suppressor-like cells; N2 neutrophils, Anti-tumor phenotype; NK, Natural killer; Tregs, Regulators T cells.

In addition to the role of natural products in PCD, they may also prevent resistance by tumor cells to conventional chemotherapeutics (Yuan et al., 2017), thanks to their intervention in tumor metabolism (Figure 3). Plant-derived components can modulate energy metabolism at both the glycolytic (Hasanpourghadi et al., 2017) and mitochondrial (Tao et al., 2019) levels in different cancer models.

Focusing on the modulation of metabolism, fermented wheat germ extract is a complex mixture of biologically active molecules with an antimetastatic activity that inhibits the enzymes glucose-6-phosphate dehydrogenase (G6PDH) and transketolase, which regulate carbon flux in glycolysis and the pathway of the pentoses in Jurkat line cell (Comi-Anduix et al., 2022). Phenolic phytometabolites such as curcumin, apigenin, resveratrol, or some alkaloids such as alkeberberine or capsaicin target HIF-1, GLUTs, or some enzymes of the glycolytic pathway such as HK-II, PFKP, and PK, among others (Hasanpourghadi et al., 2017). However, in ALs, the role of natural compounds on glycolytic metabolic regulation has been poorly evaluated.

On the other hand, phenolic compounds can act as mitocans through different mechanisms that include inhibition of HK-II, BCL-2, electron transport chain complexes, induction of oxidative stress, voltage-gated anion channel (VDAC)/adenine nucleotide translocase (ANT) complex dysregulation, or as lipophilic cations targeting the inner mitochondrial membrane, some intermediates of the TCA cycle, or mitochondrial DNA (Guerra et al., 2018). One of the widely studied anti-tumor effects of phenolic compounds is the neutralization of ROS because of its integral role in carcinogenesis and mitochondria as its main source. The antioxidant effect is attributed to the modulating capacity of intracellular antioxidant enzymes, direct elimination of ROS by donation of electrons or hydrogens, chelation of transition metals, or induction of antioxidant pathways such as Kelch-like ECH-associated protein 1- Nuclear factor erythroid 2 related factor 2 (Keap1-Nrf2) (Stevens et al., 2018). Interestingly, phenolic compounds can also exert a prooxidant role that depends on the total number of hydroxyl groups and their substitution patterns. The dual effects of some compounds are promising in combination therapies.

An example of the modulation of mitochondrial metabolism in primary AML cells is the action of lipid B extracted from avocado, which can exert a selective cytotoxic effect on leukemic cells. This specificity can be attributed to increased mitochondrial mass and altered mitochondrial metabolism. Lipid B can enter the mitochondria through carnitine palmitoyl transferase I (CPT1), an enzyme that facilitates the transport of mitochondrial lipids, favoring their accumulation and consequently the inhibition of fatty acid oxidation and reduction in NADPH levels, leading to cell death (Lee et al., 2015).

Regarding the immunomodulatory potential of natural products in ALs, in *in vitro* assays, continuous treatment of HL60 cells with DOX induces a differentiation towards type N1 neutrophils (pro-tumor phenotype); however, the addition of berberine, an alkaloid, favors a change towards type N2 neutrophils (anti-tumor phenotype) (Zhang et al., 2020). Also, chrysophanol, one of the most important anthraquinone components isolated from plants of the genus *Rheum*, was proposed as an immunomodulator by Zhong et al. (2022), since they suggested that its antitumor effects against acute leukemia T cells were due to thorough regulation of the immunosuppressive molecule PD-L1 (Yin et al., 2020). In turn, resveratrol improved T lymphocyte proliferation and NK cell activity in a mouse leukemia model (L1210) (Li et al., 2007). Further studies of natural products as immunomodulators in AL could help discover promising compounds against immune checkpoints.

The central research area of our group is the study and development of phytomedicines from Colombian plants, among them Petiveria alliacea (P. alliacea), Caesalpinia spinosa (C. spinosa), Tillandsia usneoides (T. usneoides), and Piper nigrum (P. nigrum) (Lasso et al., 2018; Urueña et al., 2020; Lasso et al., 2022; Urueña et al., 2022). Each one of the extracts of these plants has been characterized from a chemical point of view, and the main compounds have been isolated and studied. The extracts are standardized to be developed as botanical drugs due to their interesting antitumor and immunomodulatory activity in animal models (Lasso et al., 2018; Urueña et al., 2020). C. spinosa has also been studied in normal individuals to assess its safety and in coronavirus disease 2019 (COVID-19) patients, where significant biological activity has been demonstrated (Duran et al., 2022; Urueña et al., 2022).

*P. alliacea* is a plant used in Central and South America as traditional medicine due to its antispasmodic, antirheumatic, anti-inflammatory, and analgesic activities. Regarding its activity on metabolism, traditional knowledge attributes hypoglycemic properties to it (Fiorentino and Urueña, 2018). We have found cytotoxic activity in tumor cell lines of murine and human origin for melanoma, breast cancer, and acute leukemia (Sandoval et al., 2016; Prieto et al., 2019; Ballesteros-Ramírez et al., 2020b). The proteomic analysis of tumor cells treated with aqueous extract of *P. alliacea* revealed an alteration of proteins that participate in cell proliferation and energy metabolism, as well as a decrease in glucose uptake and lactate production. This suggests that the extract uses multiple biological mechanisms to regulate tumor growth (Hernández et al., 2014). In the *in vivo* model, the extract slows the progression of murine mammary tumors after orthotopic transplantation with 4T1 cells (Hernández et al., 2014).

More recently, it has been observed that the activity of *P. alliacea* extract is specific to tumor cells, inducing a reduction in the expression of the enzyme β-F1-ATPase, the concentration of intracellular ATP, and mitochondrial respiration (Hernández et al., 2017). Like other compounds obtained from plants, *P. alliacea* can act on DCs, inducing their partial activation, which is evidenced by morphological and phenotypic changes, associated with a differential profile of secreted cytokines (Santander et al., 2012). Our group is currently delving into the tumor context of acute leukemias.

On the other hand, in traditional medicine, the seeds and pods of *C. spinosa* are used to treat tonsillitis, gastric ulcers, and skin infections due to their high content of hydrolyzable tannins (derived from gallic acid) and their antibacterial, antitumor, astringent, anti-inflammatory, and healing properties. They are also known for their high antioxidant capacity (Fiorentino and Urueña, 2018). It is distributed throughout Latin America, and in Colombia, it occurs naturally. The extract obtained from *C. spinosa* was called P2Et. This extract can induce apoptosis through depolarization of mitochondria, activation of caspase 3, chromatin condensation, and decreased clonogenic capacity in K562 leukemia cells and 4T1 breast cancer cells. In addition, when used in combination with DOX at sublethal concentrations, a reduction in IC_50_ is observed, representing an increase in the net antitumor activity of the drug (Castañeda et al., 2012; Urueña et al., 2013). Interestingly, *in vitro*, P2Et is cytotoxic both in resistant lines with the expression of Pgp^+^ and in Pgp-lines in 2D and 3D cultures (Sandoval et al., 2016). It has been possible to show that the marked antioxidant activity of *C. spinosa* may participate in the regulation of ROS production inside and outside the tumor mass in some cancer models, which would confer great antitumor potential (Prieto et al., 2019). An immunomodulatory effect has been attributed to P2Et since it can induce the expression of immunogenic cell death markers such as calreticulin and High Mobility Group-Box superfamily 1 (HMGB1) and the release of ATP. In the *in vivo* model, mice transplanted with 4T1 cells treated *in vitro* with P2Et showed reduced tumor growth, and *ex vivo* analysis revealed multifunctional CD4^+^ and CD8^+^ T lymphocytes (Urueña et al., 2013; Gomez-Cadena et al., 2016).

Focusing on ALs, cells isolated from patients with ALL or AML have been shown to be less sensitive to P2Et and more sensitive to *P. alliacea*. In addition, the treatment of leukemic blasts with each of the two extracts combined with some drugs increases the sensitivity to death even in tumor cells that, in some cases, do not respond to conventional therapies. These data show that, at least *in vitro*, the extracts of *P. alliacea* and *C. spinosa* are capable of acting on human primary tumors, improving the response to chemotherapy (Ballesteros-Ramírez et al., 2020b).

Other plants with emerging anti-leukemic activity in our group are the extracts of T. usneoides and P. nigrum. In a mouse model transplanted with DA-3/ER-GM murine acute myeloid leukemia cells, both P2Et and T. usneoides-derived extracts were found to decrease the tumor load in peripheral blood with respect to the control group (unpublished data). Furthermore, in the 4T1 murine breast cancer model, extracts derived from *T. usneoides* and *P. nigrum* modulate the immune response by increasing the frequency of DC and activated CD8^+^ T cells and decreasing myeloid-derived suppressor-like cells (MDSC-LCs) and regulators T cells (Tregs) in the tumor microenvironment, thus favoring control of tumor growth (Lasso et al., 2022). At the metabolic level, *T. usneoides* and *P. nigrum* extracts behave as prooxidants in both B16-F10 melanoma cells and 4T1 breast cancer cells (Lasso et al., 2022). Regarding glucose consumption, *T. usneoides* does not induce significant changes in glucose uptake in 4T1 cells; however, it induces an evident increase at 12 h in B16-F10 cells (Lasso et al., 2022). *P. nigrum* induces a dose-dependent decrease in intracellular glucose uptake, in contrast to a small increase in intracellular glucose uptake in B16-F10 cells (unpublished data). The mechanisms related to the intrinsic sensitivity of the different tumor cells to each of these extracts could be related to differences in their metabolic plasticity, so immunomodulation and involvement in metabolic changes are being studied in the acute leukemia model. Some extracts or isolated compounds derived from plants with the capacity to induce metabolic changes and/or with immunomodulatory potential evaluated in leukemias are summarized in Table 1 and in other tumor models in Table 2.

**TABLE 1:** Summary of plant-derived extracts or compounds that target tumor metabolism or are proven immunomodulators in acute leukemias.

Extracts or compound	Activity		Mechanism	Model	Reference
	Changes metabolism	Immuno-modulation			
Wheat germ extract	X		Inhibits the enzymes G6PDH and transketolase	Jurkat cell line	Comi-Anduix et al. (2022)
Lipid B	X		CPT1-mediated accumulation within mitochondria and inhibition of fatty acid oxidation	Primary AML cells	Lee et al. (2015)
Berberine		X	Favors a change in the pro-tumor neutrophil phenotype towards an anti-tumor phenotype	HL60 cell line	Zhang et al. (2020)
Chrysophanol		X	Decreased PD-L1	Jurkat cell line	Yin et al. (2020)
Resveratrol		X	Improve T lymphocyte proliferation and NK cell activity	Mouse leukemia model (L1210)	Li et al. (2007)

CPT1, Carnitine palmitoyl transferase 1; G6PDH, Glucose-6-phosphate dehydrogenase; NK, natural killer; PD-L1, Programmed cell death ligand-1.

**TABLE 2:** Summary of some plant-derived extracts or compounds that target tumor metabolism or are proven immunomodulators in different cancer models.

Extracts or compound	Activity		Mechanism	Model	Reference
	Changes metabolism	Immuno-modulation			
Curcumin	X		Decreases the level and activity of the HK II protein, has little influence on other glycolytic enzymes (PFKP, LDH), and decreases GLUT1	Colon cancer	Vaughan et al. (2013), Wang et al. (2017)
Scutellarein	X		Decreases extracellular acidification rate and oxygen consumption rate	Breast cancer	Chen et al. (2012)
Chrysin	X		Inhibits HK-II binding to VDAC1, resulting in extensive apoptosis	Hepatocelular carcinoma	Xu et al. (2017)
Grape seed extract	X		Target ETC, complex III, deplete levels of glutathione	Head and neck cancer	Shrotriya et al. (2015)
Amorfrutin C	X		mPTP opening, mitochondrial oxygen consumption and extracellular acidification increased	Colorectal adenocarcinoma	Jacobsen et al. (2018)
P2Et	X	X	Decreases ROS, induces immunogenic cell death, depolarizes mitochondria, and increases multifunctional lymphocytes	Breast cancer Melanoma	Gomez-Cadena et al. (2016), Lasso et al. (2018), Prieto et al. (2019), Lasso et al. (2022)
*P. nigrum* extract	X	X	Decrease in intracellular glucose uptake, increases ROS, increasing the frequency of DC and activated CD8^+^ T cells and decreases MDSC-LCs and Tregs	Melanoma Breast cancer	Unpublished data
*T. usneoides* extract	X	X	Decreases intracellular glucose uptake, increases ROS, increases the frequency of DC, and activated CD8^+^ T lymphocytes, and decreases MDSC-LC and Tregs	Melanoma Breast cancer	Lasso et al. (2022)

DC, dendritic cells; ETC, electron transport chain; GLUT1, Glucose transporter 1; HK-II, Hexokinase-II; LDH, lactate dehydrogenase; MDSC-LCs, Myeloid-derived suppressor-like cells; mPTP, mitochondrial permeability transition pore; PFKP, phosphofructokinase; Tregs, Regulators T cells; VDAC1, Voltage-dependent anion-selective channel 1.

## 6 Conclusion

Both an increase in glycolytic activity and mitochondrial changes may underlie tumor cell resistance to many types of drugs. Although mechanistic evidence has been mainly in tumor cell lines, there is evidence of increased glycolytic metabolism in patients with ALs related to poor response to treatment. However, Recent *in vivo* and systemic studies in cancer patients support the notion that mitochondria, through their role in metabolic adaptation, also play a pivotal role in chemoresistance in ALs rather than the glycolytic phenotype. These findings suggest that both ways can be implicated, and possibly other factors such as population differences (genetics, lifestyle, microbiome nutrition, co-infections, oncogenic mutations, etc.) could influence this modulation and resistance to chemotherapy. Therapeutic intervention using extracts derived from plants developed as botanical medicines to induce metabolic changes by altering glycolysis or mitochondrial function in resistant leukemic cells can sensitize the tumor cell, potentiating the action of conventional chemotherapeutics and resulting in an immunomodulatory effect that allows the activation of an antitumor immune response.

## Figures and Tables

**Table:** 

α-KG	α-ketoglutarate
2-DG	2-deoxy-D-glucose
2-HG	2-Hidroxiglutarate
3-BrPA	3-bromopyruvate;
ABC transporters	ATP-binding cassette transporters
ACO2	Aconitase 2
ALs	Acute leukemias
ALDOA	Aldolase A
ALL	Acute lymphoid leukemia
AML	Acute myeloid leukemia
Ara-C	Cytarabine
ATO	Arsenic trioxide
ATP	Adenosine triphosphate
ATRA	All-trans retinoic acid
B-ALL	B-acute lymphoid leukemia; Bak: Bcl-2 homologous antagonist/killer; Bax: Bcl-2-associated X protein
BCR-ABL1	Breakpoint Cluster Region-Breakpoints in the Abelson (ABL) 1 fusion
BCL-2	B-Cell Leukemia/Lymphoma 2
CAFs	cancer-associated fibroblasts
CE	Endothelial cells
CO2	Carbon dioxide
COVID-19	Coronavirus disease 2019
CPT1A	Carnitine palmitoyl transferase 1A
CRISPR	Clustered Regularly Interspaced Short Palindromic Repeats
DC	Dendritic cells
DNR	Daunorubicin
DOX	Doxorubicin
ENO1	Enolase 1
ETC	electron transport chain
ETFB	Electron transfer flavoprotein subunit beta
FADH2	Reduced flavin adenine dinucleotide
FAO	Fatty acid oxidation
GAPDH	Glyceraldehyde-3-phosphate dehydrogenase
GLUT1	Glucose transporter 1
GPI	Glucose phosphate isomerase
GSH	Glutathione
H+	Hydrogen (proton)
HCC	Human hepatocellular carcinoma
HGMB-1	High mobility group box 1 protein
HIF-1	Hypoxia-induced factor 1
HK-II	Hexokinase II
HMGB1	High Mobility Group-Box superfamily 1
HO-1	Heme oxygenase-1
HOTAIRM1	Myeloid-specific antisense intergenic RNA - HOX transcript 1
HPDL	4-Hydroxyphenylpyruvate Dioxygenase Like
HSC	Hematopoietic stem cell
HSCT	Allogeneic stem cell transplantation
hTERT	Human telomerase reverse transcriptase
IDA	Idarubicin
IDH3A	(Isocitrate Dehydrogenase NAD+) 3 Catalytic Subunit Alpha
IDH	Isocitrate dehydrogenase
Keap1	Kelch-like ECH-associated protein 1
LDHA	Lactate dehydrogenase A
LICs	Leukemia-initiating cells
LncRNAs	Long non-coding RNAs
LND	Lonidamine
MCL1	Myeloid cell leukemia sequence 1 protein
MCT	proton-linked monocarboxylate transporter
microRNAs	Short non-coding RNAs
MDSC-LCs	Myeloid-derived suppressor-like cells
MFN-2	Mitofusin 2
MRC	Programmed cell death
MRD	Minimal residual disease
MRPL21	Mitochondrial Ribosomal Protein L21; Mitochondrial Ribosomal Protein S33
MSC	Mesenchymal cells
mtDNA	Mitochondrial DNA
NAD+	Oxidized nicotinamide adenine dinucleotide cofactor
NADPH	Reduced nicotinamide adenine dinucleotide phosphate
NCAM1	Neural cell adhesion molecule 1
NH4+	Ammonium ion
NHE1	Na (+)/H (+) antiporter
NFATC1	T-cell nuclear factor 1
Nrf2	Nuclear factor erythroid 2–related factor 2
NSG	NOD scid gamma mouse
OCR	Oxygen consumption rate
OPA1	Mitochondrial Dynamin Like GTPase
OxPhos	Oxidative phosphorylation
PARP	poly (ADP-ribose) polymerases
PBMCs	Peripheral blood mononuclear cells
PCD	Programmed cell death
PCNA	Proliferating cell nuclear antigen
PD-L1	Programmed cell death ligand-1
PDP	Pyruvate dehydrogenase phosphatase
PFKP	Phosphofructokinase
PGC1α	PPARγ coactivator-1α
PGK1	Phosphoglycerate kinase
PGMA1	Phosphoglycerate mutase 1
PKM	Pyruvate kinase 2
PPP	Pentose pathway
PTEN	Phosphatidylinositol 3,4,5-trisphosphate 3-phosphatase and dual-specificity protein phosphatase
ROS	Reactive oxygen species
SDH	Succinate dehydrogenase
SIRT	Sirtuins
SLC12A3	Sodium-dependent dicarboxylate transporter
SUCLA	Succinate-CoA ligase
T-ALL	T-acute lymphoid leukemia
TAMs	Tumor associated macrophages
TCA cycle	Cycle of tricarboxylic acids
TET2	Tet methylcytosine dioxygenase 2
TIMM8B	Mitochondrial Import Inner Membrane Translocase subunit Tim8B
TIMMDC1	Translocase of Inner Mitochondrial Membrane Domain Containing 1
TKIs	Tyrosine kinase inhibitors
Tregs	Regulatory T cells
TUG1	Taurine upregulated gene 1
UCA1	Associated with urothelial carcinoma
VDAC/ANT	Voltage-gated anion channel adenine nucleotide
