# Supplementary material for: Plant-derived extracts and metabolic modulation in leukemia: a promising approach to overcome treatment resistance
Source: Front Mol Biosci. 2023 Jul 13;10:1229760. doi: 10.3389/fmolb.2023.1229760 (PMC10382028; doi:10.3389/fmolb.2023.1229760)
Supplement: Supplementary file 1 [file Table1.docx]

**Supplemental Material**

| **Table S1.**  **Relationship between mechanisms that promote glycolysis described in acute leukemia and chemoresistance** | | | |
| --- | --- | --- | --- |
| **Factors associated with increased glycolysis** | **Association with chemoresistance** | **Model** | **Reference** |
| PI3K/AKT/mTOR Pathway | PI3K and AKT can phosphorylate ERα at Ser167 to activate ERα independently in the absence of estrogen, so the interaction between ER and PI3K/AKT/mTOR pathway hyperactivation causes breast cancer cells to lose sensitivity to endocrine therapy. | Breast cancer | Dong et al., 2021 |
| HO-1 | Promoting autophagy via PI3K/Akt pathway. | Breast cancer | Peo et al., 2018 |
| mi-RNAs and Lnc-RNAs | Regulate the expression of MDR, transcription factors, cyclins. Reduction of apoptosis, induction of autophagy, promotion of MET, alteration of drug concentrations and regulation of the related signalling pathways. | Pancreatic cancer  Breast cancer  Ovarian cancer  Leukemia  Gastric cancer | Li et al., 2021  Rahnama et al., 2022  Turk et al., 2022 |
| Lactate | Acidification of the extracellular environment leads to a decrease in DOX toxicity. | Cancer | Trebinska-Stryjewska et al., 2020 |
| HIF-1 | Expression of PDK3 which is related to inhibition of mitochondrial respiration, lactic acid production and drug resistance. | Cancer | Lu et al., 2008 |
| Expression of oncogenes including *RAS, Src, BCL-ABL,* serine/threonine kinase AKT*,* and *HER2/neu*; among others. | High expression of glucose transporters with production and release of lactate into the extracellular medium. | Cancer | Kominsky et al., 2009 |
| Mesenchymal cells | Increased expression of tetraspanins (CD9, CD81) and drug resistance proteins (BCRP, MDR1). | Breast cancer | Ullah et al., 2019 |
| BCR-ABL1: Breakpoint Cluster Region-Breakpoints in the Abelson (ABL)1 fusion; BCRP: Breast cancer resistance protein; DOX: Doxorubicin; Erα: Estrogen receptor alpha; HER2/neu: Human epidermal growth factor receptor-2 HIF-1: Hypoxia-inducible factor-1; MDR: Multiple drug resistance; MET: Mesenchyme-epithelial transition; mi-RNAs and Lnc-RNAs: Long and short non-coding RNAs, respectively; PDK3: Pyruvate dehydrogenase kinase-3; PI3K-Akt-mTOR: Phosphoinositide 3-kinase-Akt-mammalian target of rapamycin; Src: Proto-oncogene tyrosine-protein kinase | | | |
